# Congenital cytomegalovirus infection and brain injury in a newborn following maternal non-primary infection: case report of an unexpected diagnosis

**DOI:** 10.1186/s13052-025-02017-4

**Published:** 2025-06-21

**Authors:** Gregorio Serra, Ettore Piro, Deborah Bacile, Laura Antonella Canduscio, Claudia Colomba, Mario Giuffrè, Sergio Salerno, Ingrid Anne Mandy Schierz, Giovanni Corsello

**Affiliations:** 1 Department of Health Promotion, Mother and Child Care, Internal Medicine and Medical Specialties “G. D’Alessandro”, University of Palermo, Palermo, Italy; 2 Pediatric Infectious Diseases Unit, “G. Di Cristina” Children’s Hospital, University of Palermo, Palermo, Italy

**Keywords:** cCMV, Neonate, Pre-existing maternal immunity, Neurological anomalies, Brain MRI, Case report

## Abstract

**Background:**

Congenital cytomegalovirus (cCMV) infection leads to a significant burden on the health system. Relevant insights have been reached in the understanding of primary infection (PI) during pregnancy. However, knowledge gaps still exist related to maternal non-primary infections (NPI). Severe neurologic damage and hearing loss are the possible outcomes in the 17–20% of affected children. Furthermore, neither risk prevention strategies nor management are currently available for these NPI patients.

**Case presentation:**

We report on a male term newborn showing in the first days of life hyperexcitability, tremors and increased muscular tone, in addition to thrombocytopenia, initially related to an early-onset sepsis. Obstetric history revealed that the mother underwent steroid treatment during the whole first trimester of pregnancy. She had positive CMV IgG and negative CMV IgM antibodies throughout gestation. At 15 days of age, due to the persistence of neurological and hematological signs and abnormalities found on brain ultrasound (bilateral ventriculomegaly, and an anechoic lesion within the right caudothalamic grove at first related with grade I intraventricular hemorrhage) a brain magnetic resonance imaging (MRI) was performed, showing significant lesions highly suggestive of cCMV. Although such diagnostic hypothesis was unsuspected (in light of the association of clinical manifestations with perinatal sepsis and the misleading maternal serology), however CMV DNA detection on blood and urine was carried out, giving positive results in both samples for connatal infection diagnosis. Newborn CMV IgG and IgM antibodies resulted positive, while the IgG avidity test showed high values according to a likely early intrauterine infection. The antiviral therapy was therefore begun and continued for 6 months. He currently is 6 months old and included in a multidisciplinary follow-up. His growth is within the normal limits, but a neuromotor delay is present. Audiological and ophthalmological evaluations, laboratory as well as multiorgan ultrasound (US) examinations have not revealed further anomalies to date.

**Conclusions:**

Our case underlines that CMV reactivations or reinfections may be responsible, as well as PI, for significant and harmful effects on the fetus and newborn. It also shows the limited diagnostic and preventive/therapeutical weapons available against NPI during gestation. The present experience confirmed, indeed, the literature regarding the absence of valid laboratory test to identify women with preexisting immunity at risk of giving birth to an infected neonate. Women with previous immunity should be treated with precautionary protocols, including US monitoring and fetal MRI aimed at detecting cCMV. Brain MRI findings may be a pre-warning for newborns of mothers with previous immunity showing neurological symptoms and ultrasound abnormalities. In these cases, its execution may allow the identification of pathognomonic lesions.

## Background

Cytomegalovirus (CMV) is a ubiquitous DNA herpesvirus. It is the most common congenital viral agent of intrauterine infection, with a birth prevalence recently estimated at 0.64% worldwide [1]. Vertical transmission from mother to fetus can occur after either primary (PI) or non-primary (NPI) infection. The incidence of maternal PI is 1–2%, with a vertical transmission rate of 32%. Epidemiology of maternal NPI is conversely poorly documented, since its occurrence is not routinely diagnosed in pregnant women. A meta-analysis reported CMV shedding in 25.5% (95% CI, 12.7-30.3%) of seropositive pregnant women, with a vertical transmission rate probably lower than 3.5% [1, 2–3]. However, the occurrence of *sequelae* among congenital cytomegalovirus (cCMV) infected children is unrelated to the type of maternal infection [1, 4–7], but rather to the infection precociousness determining a higher risk if contracted within the first trimester of pregnancy. Therefore, fetal CMV infection should be suspected in subjects with maternal serology consistent with primary infection and/or in case of ultrasound (US) findings suggestive of intrauterine infection. In fact, a serological profile showing positive immunoglobulin G (IgG) and negative immunoglobulin M (IgM) after the 16th week of gestation cannot distinguish the type of CMV infection, since these results may still occur after either primary or non-primary first trimester maternal infection [2, 4]. The diagnosis of fetal infection is confirmed in pregnancies by CMV DNA detection through positive polymerase chain reaction (PCR), performed in the amniotic fluid by the 17th week of gestation, if the procedure is carried out at least 8 weeks after the presumed time of maternal infection [8–10].

In the newborn, viral particles should be searched in urine and/or saliva within the first 2–3 weeks of life, to distinguish congenital from postnatally acquired disease [11]. An alternative method, although with low sensitivity, is CMV DNA detection in the screening blood-spot test (Guthrie card), performed between 48 and 72 h of life [12]. Once the diagnosis is made, an effective and well-tolerated antiviral therapy (intravenous ganciclovir and oral valganciclovir) is available for symptomatic infants to improve hearing and neurodevelopmental outcomes.

## Case presentation

The patient is a male newborn, first child of non-consanguineous parents. He was delivered at 38^+ 4^ weeks of gestation (WG) by caesarean section, due to flowmetric alterations (abnormally high umbilical artery pulsatility index [PI > 2 standard deviations, SD] associated with a significantly low PI [< 5th centile] of the fetal middle cerebral arteries) and anhydramnios [13]. Pregnancy history revealed that the 36-year-old mother was a tobacco user, smoking 20 cigarettes a day. Moreover, she underwent steroid treatment (oral prednisone 12.5 mg/day) during the whole first trimester of gestation for repeated miscarriages, due to antiphospholipid syndrome unresponsive to first line drugs (acetylsalicylic acid and low-molecular-weight heparin). During the previous pregnancy, occurred four years before and resulted in miscarriage, the mother had high CMV specific IgG levels (372 IU/ml, cut-off > 1). Within the first trimester of the current pregnancy, CMV IgG antibodies resulted positive (> 500 IU/ml, cut off > 1) as well, although with higher values than previously detected in the same laboratory, to which however in that moment the care team did not focus on. Such little weight was given also due to the concurrent evidence of negative IgM antibodies (0.149 IU/ml, cut-off < 0.7). In the second trimester, IgG antibodies were positive (500 IU/ml) and IgM still showed negative values (0.152 IU/ml, cut off < 0.7). Also in the last trimester IgM antibodies were repeated, and a negative profile was finally observed (0.178 IU/ml).

**Fig. 1:**
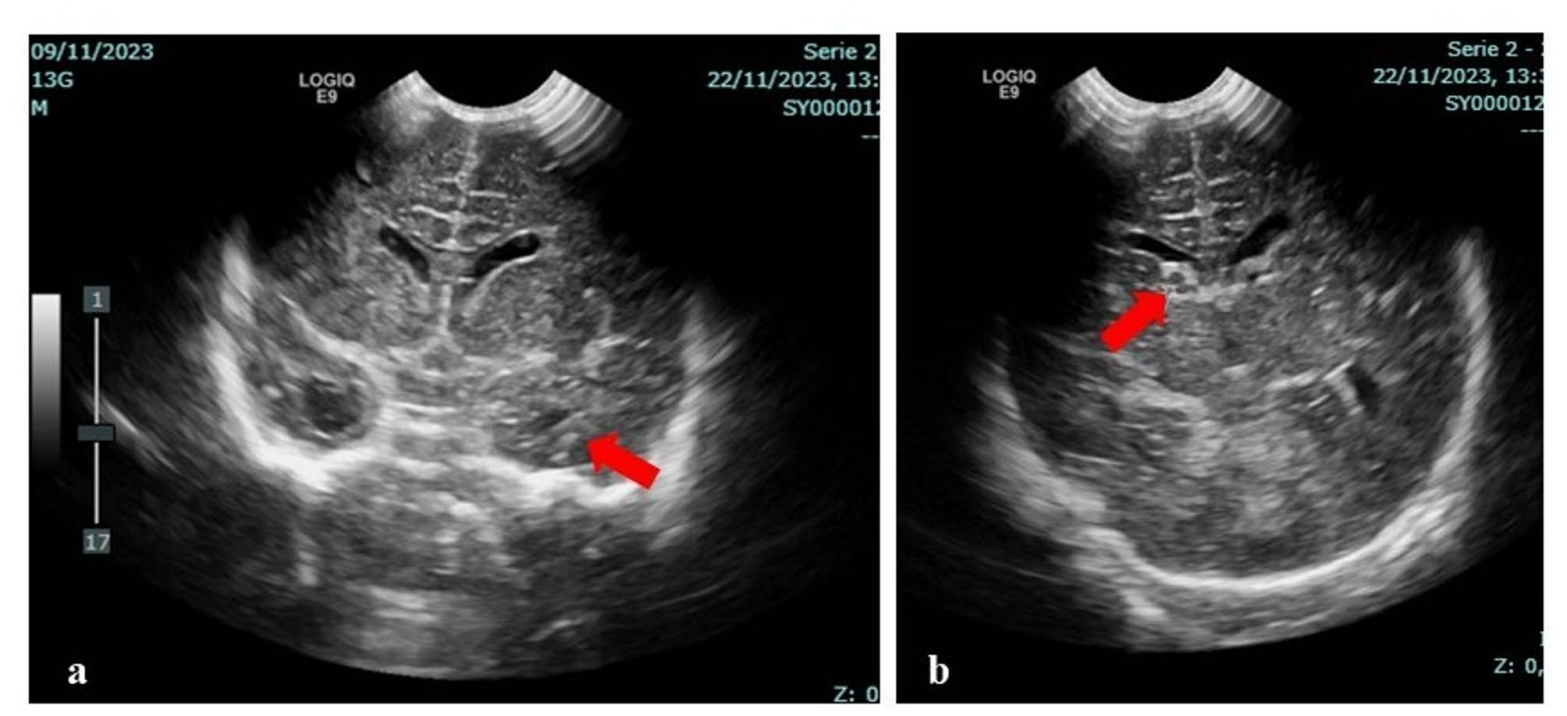
Transfontanellar US at 10 days of life: **a** bilateral and asymmetric ventriculomegaly due to dilatation of the temporal horns, with left prevalence; **b** anechoic lesion within the right caudothalamic groove, indicated by arrow

The serological tests for the other TORCH pathogens were negative. Prenatal US evaluations, performed in the first and second trimester of pregnancy, did not reveal any abnormalities, while the US of the third one documented, indeed, a dilation of both lateral ventricles. A II level in-depth investigation, through fetal magnetic resonance imaging (MRI), although requested by obstetricians, was imprudently refused by the couple, as the evolution of this clinical history will later document. Vaginal and rectal swabs for Streptococcus *agalactiae* were unknown. At birth, adaptation to extrauterine life was normal, with Apgar scores 10 at 1 and 5 min. Anthropometric measures were as follows: weight 2750 g (12th centile, -1.17 SD), length 48 cm (19th centile, -0.86 SD), occipitofrontal circumference (OFC) 33.5 cm (27th centile, -0.62 SD), according with the Italian Ines Growth Charts [14]. Postnatally, he presented with a dystrophic appearance, with poor generalized presence of body adipose tissue. Neurological examination revealed hyperexcitability with spontaneous and post-stimulation tremors, along with an increased generalized muscular tone. In the first day of life, due to mild respiratory distress associated with feeding difficulties, he required oxygen therapy and parenteral nutritional support for about 24 and 78 h respectively. Laboratory tests documented, after 24 h, relative neutropenia (white blood cells 8,850/mm^3^; neutrophils 1,460/mm^3^, 16.5%; lymphocytes 6,060/mm^3^ 68.5%), thrombocytopenia (92,000/mm^3^), and high levels of c-reactive protein (CRP 33 mg/dl, normal values < 5) according with an early-onset sepsis, later confirmed by Group-B streptococcus growth revealed on blood culture. Therefore, antibiotic therapy with intravenous (i.v.) ampicillin and gentamicin was started (the latter stopped shortly after), leading to progressive clinical improvement and of hemochromocytometric parameters, and normalization of inflammatory markers within one week. During the first days of life, he underwent multiorgan US evaluations. Head US revealed a microcavitation within the right caudothalamic groove, raising the suspicion of grade I intraventricular hemorrhage (IVH), as well as irregular profile of the choroid plexuses and bilateral periventricular hyperechogenicity; heart US showed patent *foramen ovale* and *ductus arteriosus*, in addition to tricuspid regurgitation. Abdominal US showed a mild splenomegaly with maximal splenic length 6.2 cm, considering 6 cm as the upper normal limit for age-matched healthy subjects (the width was assessed at the hilum on a longitudinal coronal image, according to the American Society for Pediatric Radiology recommendations [15]). For such spleen involvement, anyhow, no further pathogenetic association was made, as thereafter will be clearer, besides that with perinatal sepsis. Transfontanellar US was repeated at 10 days of life, revealing bilateral and asymmetric ventriculomegaly due to dilatation of the temporal horns (with left prevalence) (Fig. 1a), and confirming the anechoic lesion within the right caudothalamic groove (Fig. 1b), which was apparently in accordance with the initial hypothesized picture of I grade IVH.

**Fig. 2:**
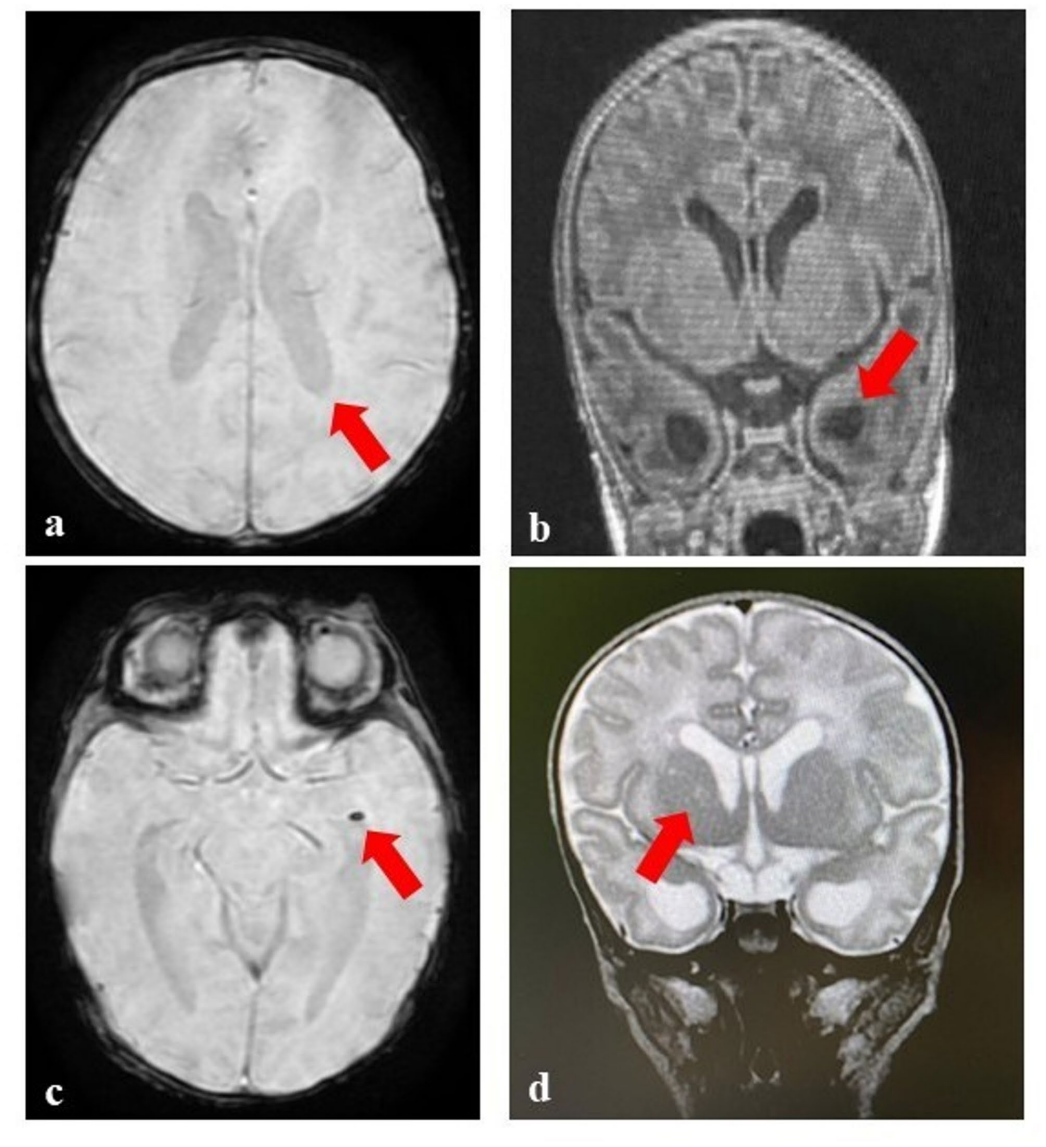
Brain MRI. **a** Lateral ventricles dilation; **b** cystic lesions within the ependyma of the temporal horns; **c** millimetric calcification in the left ventricular ependyma; **d** lacunar lesion within the right thalamus. All findings are indicated by arrows

On day 15, owing to the persistence of thrombocytopenia (79,000/mm^3^) with decreased values of mean platelet volume (MPV, 6.7 fL, normal values 7.4–10.4), and of the neurological signs previously highlighted, in addition to the not univocally interpretable anomalies detected on cerebral US, a brain MRI has been performed. It confirmed the lateral ventricles dilation (Fig. 2a); however, in addition, it surprisingly defined the presence of cystic lesions within the ependyma of the temporal horns (Fig. 2b), as well as a millimetric calcification in the left ventricular ependyma (Fig. 2c) along with a lacunar lesion within the right thalamus (Fig. 2d).

Such cerebral abnormalities, only at this point were connected by neonatologists with the other aspecific and/or misinterpreted (as related with the perinatal sepsis), clinical (dystrophic appearance, hyperexcitability, tremors, increased muscular tone), hematological (thrombocytopenia) and instrumental (splenomegaly) findings, raising the hypothesis of congenital CMV infection. Therefore, due to these unexpected data and the linked diagnostic suspicion, unconsidered until then also in light of the misleading maternal serological profile, CMV DNA detection on blood and urine was carried out. It gave positive results in both samples, with viruria (43,783,755 genomic copies/ml) extremely higher than viraemia (1,337 genomic copies/ml), for congenital CMV infection diagnosis.

Given the impossibility of precisely dating the non-primary maternal infection, the IgG and IgM serological profile along with the IgG avidity test (despite being aware of the limits of this method [16, 17]) were performed on the newborn and the mother after birth. The newborn’s serology showed positive CMV IgG with high avidity of IgG antibodies, according to a likely first/second trimester transmission (confirmed also in a different laboratory one week later). The avidity analysis, in addition to the clinical data, guided us in ruling out a recent third trimester infection. The complete mother and newborn microbiological profiles are summarized in Table 1.

**Table 1:** Microbiological profiles of mother and newborn

	Mother, I trimester of pregnancy	Mother, II trimester of pregnancy	Mother, III trimester of pregnancy	Mother after delivery	Newborn (15 days of life)
**CMV IgG: **	**Positive** (> 500 IU/ml, cut off > 1)	**Positive** (500 IU/ml, cut off > 1)	n.p.	**Positive** (> 180 AU/ml, cut-off > 14)	**Positive** (153 AU/ml, cut-off > 14)
**CMV IgM**:	**Negative** (0.149 IU/ml, cut off < 0.7)	**Negative** (0.152 IU/ml, cut off < 0.7)	**Negative** (0.178 IU/ml, cut off < 0.7)	**Negative** (17.3 AU/ml, cut-off > 22)	**Positive** (40.3 AU/ml, cut-off > 22)
**CMV IgG AVIDITY**	n.p.	n.p.		**High** (0.86; cut-off ≥ 0.65; intermediate 0.40 ≤ value < 0.65; low < 0.40)	**High** (0.76, cut-off ≥ 0.65; intermediate 0.40 ≤ value < 0.65; low < 0.40)
**Immunoblotting IgG/IgM/IgA**				**Present/Present**	**Present/Absent/Absent**
**CMV DNA urine**					43,783,755 genomic copies/ml
**CMV DNA blood**					1,337 genomic copies/ml

n.p. = not performed

In the meanwhile, owing to the persistence of mild feeding difficulties, intravenous therapy with Ganciclovir (6 mg/kg q12hr) was started, and administered for 7 days. Subsequently, oral Valganciclovir (16 mg/kg q12hr) was given [18–20] and continued for overall 6 months, according to the 2017 consensus recommendations by Rawlinson WD et al. [21], and the following ones for prenatal, neonatal and postnatal management of congenital cytomegalovirus infection by the European congenital infection initiative [8].

Hearing screening through transient-evoked otoacoustic emissions (TEOAEs), as well as auditory brainstem response (ABR) were normal. Visual evoked potentials and ophthalmological evaluation showed normal results as well. He was discharged at 28 days of life, and included in a multidisciplinary (auxological, neurodevelopmental, ophthalmological, audiological) follow-up. He currently is 6-month-old, with anthropometric measures as follows: weight 8200 g (59th centile, + 0.23 SD), length 68 cm (52nd centile, + 0.06 SD), occipitofrontal circumference (OFC) 43 cm (36th centile, -0.36 SD) according to World Health Organization growth charts [22]. The developmental assessment shows a normal response threshold to sound stimuli (bell and rattle), with orientation to human voice. He can focus on the red cube and yellow duck, but not on a small object. He is able, held in a supported sitting position, to grab from the table the red cube and the yellow duck with both hands on the median line with oral exploration. In the supine position there is a slight postural instability with abduction of the upper limbs, and voluntary medialization movements of arms are evoked with the ring suspended in front of him. He shows a mild hypertonia of the limbs with hyperreflexia, and normal angle of dorsiflexion of the ankle bilaterally. A moderate deficit of lifting on the arms in prone position is present. He does not show any further clinical anomalies. Both laboratory tests as well as US multiorgan and neurosensorial evaluations do not put in evidence other abnormalities to date.

## Discussion and conclusions

Congenital CMV infection is the most common congenital infection worldwide. It is responsible for a significative burden on health care system, due to a global prevalence at birth estimated at 0.64%, and risk of severe long-term adverse outcomes (sensorineural hearing loss and neurodevelopmental delay) of 17–20% among infected children. Compared to the previous indications provided by the consensus published in 2017 [21], the understanding and knowledge about PI improved over recent years [8]. Conversely, the question about non-primary infections and their management, as in our case, is still object of open debate. Indeed, while in PI the antiviral treatment with valaciclovir has been proven to be effective for the prevention of vertical transmission, the diagnostic and prophylactic/therapeutical weapons against NPI during gestation are still limited. Nonetheless, the scientific community is now significantly more aware that the risk of serious *sequelae* is associated with early maternal infections regardless of the type of infection (NPI or PI) [8, 23–26]. Indeed, cCMV can occur after primary or non-primary maternal infections. Our case falls within the latter *scenario*, and confirms that efforts are still needed to better manage infections during pregnancy, as also demonstrated by the neurological outcomes of the present patient. Actually, there is no valid laboratory test to identify women with preexisting immunity at risk of giving birth to an infected neonate [27, 28]. CMV serology and PCR are not helpful, with IgM detection rate being reported in 0–25% of these women, a rise in IgG titers between 0 and 22%, and positive DNAemia in 24–66% of them [8]. Careful prenatal ultrasound monitoring with serial targeted assessments, as well as the execution of a fetal MRI in the third trimester, remain, then, our main tools, as they provide relevant information for diagnosis and prognosis. In our case, anomalies were detected on the prenatal ultrasound imaging, which, although non-specific, led the gynecologists to require an MRI; however, it was refused by the couple. The evidence and a more in-depth confirmation of neurological findings suggestive of cCMV on fetal MRI could have directed neonatologists to carry out CMV-DNA research on urine, saliva and blood already in the first days of life [29]. The negative predictive value for moderate/severe *sequelae* of normal fetal MRI is close to 100%, with a residual risk of 17% of bilateral deafness [30–32].

The present report highlights the relevance of paying attention to any neonatal clinical manifestations consistent with cCMV. Actually, our newborn showed neurological symptoms, mild splenomegaly and thrombocytopenia, in addition to ultrasound abnormalities, which, however, could also be due to the culture-proven Group B Streptococcus early onset sepsis. This further underlines how difficult is, to date, to interpret mild or isolated clinical or laboratory characteristics as signs compatible with cCMV (in fact those of our newborn, before performing MRI, were non-specific), which can justify the execution of a postnatal test for cCMV diagnosis in the presence of a known preconception maternal immunity. Furthermore, this condition must be distinguished by other ones showing overlapping clinical signs like congenital defects, autoimmune diseases, sepsis, hypoxic-ischemic encephalopathy, hereditary metabolic disorders or connatal infections sustained by TORCH agents different than CMV [33–46]. It is hard, as well, to establish whether the maternal steroid therapy carried out in the first trimester of pregnancy could have played a role in determining a condition of immunosuppression leading to CMV reactivation/reinfection. In this regard, poor literature data are available to support such hypothesis [47, 48]. This case underscores how congenital CMV (cCMV) infection resulting from maternal non-primary infection, although less frequently, can be as severe as cCMV following maternal PI. In our newborn, the laboratory investigations performed at birth showed that the infection was probably contracted in an early stage of gestation (first/second trimester), and this led to severe *sequelae*. A synopsis comparing our patient to those born to mothers with NPI recently reported in the literature is presented in Table 2.

**Table 2:** Comparison between our patient and those reported in the literature born to mothers with non-primary infection

Authors	Arnouts L et al. (2022) [49]	Gunkel J et al. (2017) [50] Patient 1	Gunkel J et al. (2017) [50] Patient 2	Gunkel J et al. (2017) [50] Patient 3	Our patient
**NPI CMV IgG/IgM (Mother)**	+	+/-	+/-	+/-	+/-
**Alterations on prenatal US (third trimester pregnancy)**	n.r.	Fetal echogenic bowels and bilateral ventriculomegaly	Bilateral ventriculomegaly	Echogenic bowels and oligohydramnios	Bilateral ventriculomegaly, anhydramnios
**Birth weight**	SGA	AGA	AGA	AGA	AGA
**Haematological abnormalities**	Thrombocytopenia with petechiae	Thrombocytopenia with petechiae	Thrombocytopenia	Thrombocytopenia with petechiae	Thrombocytopenia
**Liver dysfunction**	Hypertransaminasemia	n.r.	n.r.	Conjugated hyperbilirubinemia	Normal liver function
**Neurological anomalies**	Hypotonia	n.r.	n.r.	n.r.	Tremors, hyperexcitability, increased axial and limbs muscular tone
**Hepatomegaly/Splenomegaly**	**+/**-	**+/+**	n.r.	n.r.	-/+
**US findings**	n.r.	Ventricular dilatation, white matter calcifications, and bilateral germinolytic cysts (1 day of life)	Bilateral ventriculomegaly, germinolytic cysts, and a cyst in the right temporal lobe (1 day of life)	Mild bilateral ventriculomegaly and a smooth aspect of the cortex (1 day of life)	Microcavitation within the caudothalamic groove (1 day of life); bilateral ventriculomegaly, anechoic lesion within the right caudothalamic groove (10 days);
**MRI findings**	Ventricular dilatation and germinolytic cysts	White matter signal intensity changes and resolution of the germinolytic cysts (5 months of age)	Germinolytic cysts, subependymal cysts, large temporal cysts, and small occipital cysts (2 day of life)	Extensive polymicrogyria, intraventricular hemorrhage, subdural hemorrhage, supra- and infratentorial hemorrhagic lesions in the white matter, and multiple punctate hemorrhages in the cerebellum (3 day of life)	Lateral ventricles dilation, cystic lesions within the ependyma of the temporal horns, millimetric calcification in the left ventricular ependyma, lacunar lesion in the right thalamus (15 day of life)
**ABR/VEP**	Abnormal/n.r.	Normal/Normal	Normal/Normal	n.r. / abnormalities suggestive of chorioretinitis	Normal/Normal
**Treatment with Valganciclovir**	+	+	+	n.r.	+
**Follow-up**	n.r.	Mild neurodevelopmental impairment (12 months of age)	Normal neurological development (36 months of age)	Death after a few days	Moderate degree neuromotor delay, mild hypertonia

ABR = Auditory Brainstem Response; AGA = adequate for gestational age; SGA = small for gestational age; VEP = Visual Evoked Potential; n.r. = not reported

Present experience confirms the literature regarding the absence of valid laboratory tests to identify women with preexisting immunity at risk of giving birth to an infected neonate The adoption of universal screening program for neonates at birth, currently object of open debate, could contribute to early identification and prompt treatment of all affected subjects. In the meanwhile, women with previous immunity should be treated with precautionary protocols, including preventive hygiene standards, along with I and II level imaging monitoring aimed at detecting cCMV. Brain MRI may be a pre-warning for those born to mothers with previous immunity, showing neurological symptoms and ultrasound abnormalities. In these cases, indeed, its execution may allow the identification of central nervous system pathognomonic lesions (as documented also in our patient), and then to address the correct diagnostic path. Once raised the clinical suspicion, specific microbiological and hematochemical exams, in addition to supportive multiorgan US and specialistic (ophthalmological, audiological) evaluations, should be promptly started to reach the diagnosis and begin an individualized management. This must include both proper therapy and adequate multidisciplinary (involving neonatologists, obstetricians, microbiologists, infectious disease specialists, radiologists, surgeons, pediatric neurologists and psychologists, audiologists, ophthalmologists) follow-up, oriented to improve sensorial and neurodevelopmental outcomes, and then to a better expectancy and quality of life for patients and their families. Finally, given the known short and long-term clinical consequences, careful longitudinal surveillance of children with congenital CMV infection should be recommended at least until school age, and tailored on a case-by-case basis.

## Data Availability

The datasets used and analyzed during the current study are available from the corresponding author on reasonable request.

## Abbreviations

cCMV: Congenital cytomegalovirus
IgG: Immunoglobulin G
IgM: Immunoglobulin M
IVH: Intraventricular hemorrhage
MRI: Magnetic resonance imaging
NPI: Non primary infection
PI: Primary infection
US: Ultrasound
